# Effectiveness of vortioxetine in real-world clinical practice: Interim results from the relieve study

**DOI:** 10.1192/j.eurpsy.2021.915

**Published:** 2021-08-13

**Authors:** G. Mattingly, M. Christensen, K. Simonsen, L. Hammer-Helmich, H. Ren

**Affiliations:** 1 St. Charles Psychiatric Associates, Midwest Research Group, Saint Charles, United States of America; 2 Medical Affairs, H.Lundbeck A/S, Valby, Denmark; 3 Value Evidence Analytics, H.Lundbeck A/S, Valby, Denmark; 4 Value Evidence, Medical Affairs, H.Lundbeck A/S, Valby, Denmark

**Keywords:** real world evidence, effectiveness, Depression, Vortioxetine

## Abstract

**Introduction:**

Vortioxetine has demonstrated sustained efficacy and favorable safety profile in multiple clinical trials.

**Objectives:**

This study aims to describe the effectiveness and safety of vortioxetine in real-world clinical practice.

**Methods:**

RELIEVE is a prospective, multi-national, observational cohort study of outpatients initiating vortioxetine treatment for MDD at physician’s discretion and followed for 6 months. Data were collected at routine clinical visits. The primary outcome was functioning measured by Sheehan Disability Scale (SDS). Depressive symptoms measured by Patient Health Questionnaire 9-item (PHQ-9), cognitive symptoms measured by PDQ-5 and DSST were key secondary outcomes. Safety outcomes including adverse events were reported. This interim analysis presents results of 527 patients who completed the study and were followed for 6 months. Mixed models of repeated measures were used to assess improvements between baseline and month 6, adjusted for relevant confounders.

**Results:**

A total of 527 patients (mean age, 50.2 years, 65% female) were enrolled from US, Canada, France and Italy, and included in the analysis. Mean SDS total score, PHQ-9, PDQ-5 scores decreased by 8.6, 7.4 and 4.7 respectively from baseline to last visit. Mean DSST score improved by 6.5 from baseline to last visit. Patients’ overall functioning and quality of life significantly improved, sick leave days and underproductive days (both absenteeism and presenteeism) decreased over the entire follow up period. The overall incidence of adverse events(AE) was 25%, with the most common AEs being nausea and headache.

**Conclusions:**

The results confirm the effectiveness and good tolerability of vortioxetine in a broad range of patients in routine clinical practice.

**Conflict of interest:**

Dr. Mattingly has served as researcher, consultant or speaker for Akili, Alcobra, Alkermes, Allergan, Axsome, Boehringer, Forum, Genentech, Jansen, Lundbeck, Medgenics, Merck, Neos, NLS Pharma, Otsuka, Reckitt Benckiser, Roche, Sage, Shire, Sunovion, Supe

